# Dissecting the Impact of Vascular Smooth Muscle Cell ABCA1 versus ABCG1 Expression on Cholesterol Efflux and Macrophage-like Cell Transdifferentiation: The Role of SR-BI

**DOI:** 10.3390/jcdd10100416

**Published:** 2023-10-02

**Authors:** Olanrewaju Oladosu, Ikechukwu C. Esobi, Rhonda R. Powell, Terri Bruce, Alexis Stamatikos

**Affiliations:** 1 Department of Food, Nutrition, and Packaging Sciences, Clemson University, Clemson, SC 29634, USA; oolados@g.clemson.edu (O.O.); iesobi@g.clemson.edu (I.C.E.); 2 Clemson Light Imaging Facility, Clemson University, Clemson, SC 29634, USA; rhondar@clemson.edu (R.R.P.); terri@clemson.edu (T.B.)

**Keywords:** atherosclerosis, dedifferentiation, lipid, lipoprotein, reverse cholesterol transport

## Abstract

Cholesterol-laden macrophages are recognized as a major contributor to atherosclerosis. However, recent evidence indicates that vascular smooth muscle cells (VSMC) that accumulate cholesterol and transdifferentiate into a macrophage-like cell (MLC) phenotype also play a role in atherosclerosis. Therefore, removing cholesterol from MLC may be a potential atheroprotective strategy. The two transporters which remove cholesterol from cells are ABCA1 and ABCG1, as they efflux cholesterol to apoAI and HDL, respectively. In this study, the well-characterized immortalized VSMC line MOVAS cells were edited to generate ABCA1- and ABCG1-knockout (KO) MOVAS cell lines. We cholesterol-loaded ABCA1-KO MOVAS cells, ABCG1-KO MOVAS cells, and wild-type MOVAS cells to convert cells into a MLC phenotype. When we measured apoAI- and HDL-mediated cholesterol efflux in these cells, we observed a drastic decrease in apoAI-mediated cholesterol efflux within ABCA1-KO MOVAS MLC, but HDL-mediated cholesterol efflux was only partially reduced in ABCG1-KO MOVAS cells. Since SR-BI also participates in HDL-mediated cholesterol efflux, we assessed SR-BI protein expression in ABCG1-KO MOVAS MLC and observed SR-BI upregulation, which offered a possible mechanism explaining why HDL-mediated cholesterol efflux remains maintained in ABCG1-KO MOVAS MLC. When we used lentivirus for shRNA-mediated knockdown of SR-BI in ABCG1-KO MOVAS MLC, this decreased HDL-mediated cholesterol efflux when compared to ABCG1-KO MOVAS MLC with unmanipulated SR-BI expression. Taken together, these major findings suggest that SR-BI expression in MLC of a VSMC origin plays a compensatory role in HDL-mediated cholesterol efflux when ABCG1 expression becomes impaired and provides insight on SR-BI demonstrating anti-atherogenic properties within VSMC/MLC.

## 1. Introduction

Atherosclerosis is a chronic inflammatory condition caused by cholesterol accumulating in arteries [1,2]. While the accumulation of cholesterol can occur in arterial endothelial cells and vascular smooth muscle cells during atherosclerosis, sub-endothelial macrophage cholesterol accumulation is recognized as the main driver of atherosclerosis [3,4,5,6]. Intimal lipid-laden macrophages are considered a hallmark of atherosclerosis [7,8] and so research has been focused on treatments that prevent or reverse cholesterol accumulating within intimal macrophages [9,10]. However, more recent data suggests most cholesterol-laden intimal cells are of vascular smooth muscle cell (VSMC) origin [11,12]. The disagreement over VSMC versus macrophage intimal foam cell populations arises from the cholesterol accumulation in VSMC causing VSMC to transdifferentiate into a macrophage-like cell (MLC) [13,14]. Therefore, uncovering alternative therapies geared toward reducing cholesterol content within intimal VSMC/MLC, in light of the current knowledge, may be an appropriate approach for treating atherosclerosis.

The removal of cellular cholesterol is diverse among cell types, but two transporters which regulate cholesterol efflux in macrophages and other peripheral cells are the ATP-binding cassette transporter A1 (ABCA1) and the ATP-binding cassette transporter G1 (ABCG1) [15]. ABCA1 is crucial to reversing cholesterol transport because ABCA1 is required for apoAI-mediated cholesterol efflux, which results in generating nascent HDL [16,17,18,19]. ABCG1 is also vital to reversing cholesterol transport by participating in HDL-mediated cholesterol efflux [20]. Both transporters are considered atheroprotective by contributing to reverse cholesterol transport and major cellular cholesterol efflux pathways [21,22]. There is a wealth of data to imply macrophage ABCA1 and ABCG1 expression is atheroprotective [23]. In comparison, published data is scant regarding whether VSMC ABCA1/ABCG1 expression is anti-atherogenic.

Scavenger receptor B1 (SR-BI) is capable of promoting cholesterol efflux to HDL particles [22]. Like ABCA1 and ABCG1, much of the published literature on SR-BI-mediated cholesterol efflux has focused on its expression in macrophages. Remarkably, SR-BI can participate in cholesterol influx [24,25], particularly within hepatocytes [22]. This dual-function of SR-BI makes it crucial for regulating whole-body cholesterol metabolism [25]. HDL binding to endothelial SR-BI is shown to stimulate atheroprotective nitric oxide [26,27]. However, endothelial cell SR-BI facilitates LDL transcytosis, which promotes atherogenesis [28]. Therefore, it is possible that SR-BI expression in vessel wall cells exhibits both atherogenic and atheroprotective properties, though it is poorly understood whether VSMC/MLC SR-BI expression is either atheroprotective or atherogenic.

The primary purpose of this work was to attempt to uncover the atheroprotective roles of ABCA1 and ABCG1 expression in cultured VSMC. For our study, we utilized the well-characterized immortalized mouse aortic smooth muscle cell line MOVAS cells [29,30,31] for the generation of ABCA1 knockout (A1-KO) MOVAS cells, ABCG1 knockout (G1-KO) MOVAS cells, and ABCA1/ABCG1 double-knockout (DKO) MOVAS cells to compare to parental wild-type (WT) MOVAS cells. Using these cells, we tested the hypothesis that ABC transporter expression restores the VSMC phenotype in MLC by preserving apoAI/HDL-mediated cholesterol efflux. Interestingly, our results show HDL-mediated cholesterol efflux is still maintained in ABCG1-deficient MLC through SR-BI upregulation, which suggests that SR-BI plays a compensatory role in regulating HDL-mediated cholesterol efflux in VSMC/MLC when ABCG1-dependent cholesterol efflux is impaired.

## 2. Materials and Methods

### 2.1. Cell Culture and Maintenance of Cells

The immortalized vascular smooth muscle cell line MOVAS cells [29,31] were obtained from American Type Culture Collection (Manassas, VA, USA). Wild-type (WT) MOVAS cells were used to produce ABC transporter knockout (KO) MOVAS cells via CRISPR-Cas9 and gene editing was completed as a fee-for-service by the Genome Engineering & Stem Cell Center (GESC@MGI) at Washington University in Saint Louis [30]. To generate ABCA1 KO (A1-KO) MOVAS cells, a synthetic gRNA with the spacer sequence 5′-GAATGGGCAATTCGCAAACT was obtained from Integrated DNA Technologies (Coralville, IA, USA) and complexed with recombinant Cas9 protein acquired from the MacroLab at QB3-Berkeley before being nucleofected (Lonza, Basel, Switzerland) into cells. To produce ABCG1 KO (G1-KO) MOVAS cells, the same strategy described above was utilized, with the synthetic gRNA spacer sequence used being 5′-TCATGGGTCCTTCTGGAGCT. Nucleofected MOVAS cells were subsequently single cell sorted into 96-well plates and single clones were screened via next-generation sequencing for harboring only out-of-frame indels within respective alleles. ABCA1/ABCG1 double KO (DKO) MOVAS cells were produced by knocking out ABCG1 from a clone of the A1-KO cells and parental WT MOVAS cells were used as control cells in our studies. All cells were cultured using standard growth medium [29] containing high-glucose Dulbecco’s Modified Eagle’s Medium (DMEM; Corning, New York, NY, USA) supplemented with penicillin–streptomycin (P/S; 1%; Corning), G418 (500 μg/mL; VWR Life Science, Radnor, PA, USA), and FBS Essence (10%; VWR Life Science). MOVAS were maintained in 10 cm tissue culture (TC) dishes and incubated at 5% CO_2_ and 37 °C. Cells were rinsed with PBS and replenished with standard growth medium every 2–3 days until they were used for experimental studies. Prior to conducting these studies, cells reached a confluency of approximately 80% within each treatment plate.

### 2.2. Cellular Treatments

We used an established protocol to induce VSMC-to-MLC transdifferentiation in MOVAS cells [13,14]. Briefly, we cultured MOVAS cells in DMEM containing 1% P/S, 2 mg/mL of fatty acid-free bovine serum albumin (BSA-FAFA; Sigma-Aldrich, St. Louis, MO, USA), and 10 μg/mL of cholesterol–methyl-β-cyclodextrin (MβCD:Chol; Sigma-Aldrich) for 72 h to induce MLC transdifferentiation. We also treated a subset of MOVAS cells in similar conditions but without MβCD:Chol. To attempt to restore the VSMC phenotype in MOVAS MLC, we rinsed cells with PBS, then treated cells for 72 h using medium containing DMEM, 1% P/S, 2 mg/mL BSA-FAFA, and one of the following cholesterol acceptors: 100 μg/mL apoAI (Academy Bio-Medical Company, Houston, TX, USA); 100 μg/mL HDL (Academy Bio-Medical Company); or human serum (2.5%; Sigma-Aldrich). We also incubated a subset of MOVAS cells in similar conditions to those described above but replaced cholesterol acceptors with vehicle only. To manipulate SR-BI expression in MOVAS cells, we transduced cells with a lentiviral vector that expresses shRNA directed at SR-BI (VectorBuilder, Chicago, IL, USA). For the control group of MOVAS cells, we transduced cells with a lentiviral vector expressing a non-targeted scrambled shRNA (VectorBuilder). The multiplicity of infection (MOI) used for all experiments involving lentiviral transduction was 5. Cells were transduced for 24 h, rinsed with PBS, and then used for downstream experiments.

### 2.3. Immunoblotting

WT, A1-KO, G1-KO, and DKO MOVAS cells were cultured in 6-well TC plates. For experiments that assessed ABCA1 and ABCG1 expression, we exposed cells to the LXR agonist GW3965 (10 μM; Sigma-Aldrich) [32] for 24 h. The rationale for GW3965 treatment was to assess the possibility of any unedited cells still remaining in our KO cell populations which may be revealed by treating cells with GW3965, since this agonist robustly upregulates ABCA1/ABCG1 expression [32,33]. After GW3965 treatments, cells were rinsed with PBS, and harvested using RIPA lysis buffer and protease inhibitors (VWR Life Science) [34]. We quantified cellular protein using a BCA assay kit (BioVision, Milpitas, CA, USA) and an equal mass of protein for each sample was separated via SDS-PAGE, followed by PVDF membrane transfer (Merck Millipore Ltd., Burlington, MA, USA). After the protein transfer, we blocked PVDF membranes, and probed proteins of interest using the following primary antibodies: mouse anti-ABCA1 (1:1000 dilution, sc-58219; Santa Cruz Biotechnology, Dallas, TX, USA); rabbit anti-ABCG1 (1:5000 dilution, NB400-132; Novus Biologicals, Littleton, CO, USA); mouse anti-SR-BI (1:2000 dilution, MABC730; Sigma Aldrich); mouse anti-GAPDH (1:1000 dilution, sc-365062; Santa Cruz Biotechnology); and mouse anti-HSP90 (1:5000 dilution, 610419; BD Biosciences, San Jose, CA, USA). GAPDH and HSP90 were chosen as loading controls (i.e., housekeeping proteins) and selection of which representative probing result to use was based on attempting to prevent high background noise yet still detecting an optimal signal while limiting signal saturation [35]. After primary antibody incubation, we incubated PVDF membranes with one of the following secondary antibodies: HRP-conjugated goat anti-mouse IgG secondary antibody (1:10,000 dilution, AP181P; Sigma-Aldrich); HRP-conjugated rabbit anti-mouse IgM (1:10,000 dilution, SAB3701199; Sigma Aldrich); or HRP-conjugated goat anti-rabbit IgG (1:10,000 dilution, HAF008; Novus Biologicals). Afterwards, we used ECL (Immobilon ECL Ultra Western HRP Substrate; MilliporeSigma, Billerica, MA, USA) to detect HRP-conjugated secondary antibodies and imaging was performed with a ChemiDoc system (Analytik Jena US, Upland, CA, USA).

### 2.4. Cholesterol Efflux Assays

MOVAS cells were seeded into 48-well TC plates and treated using DMEM supplemented with BSA-FAFA (2 mg/mL), P/S (1%), [^3^H] cholesterol (1 μCi/mL; PerkinElmer, Waltham, MA, USA), and MβCD:Chol (10 μg/mL) [13,14], for 72 h. After treatments, we rinsed cells with PBS and replenished cells with DMEM:P/S:BSA-FAFA containing either vehicle only or one the following cholesterol acceptors: ApoAI (100 μg/mL); HDL (100 μg/mL); or human serum (2.5%). After treating MOVAS cells for 72 h, we filtered the conditioned medium to remove non-adherent cells. We also rinsed MOVAS cells with PBS and lysed cells using sodium hydroxide. We counted [^3^H] within the cellular extracts and conditioned medium using a PerkinElmer Tri-Carb 4910TR liquid scintillation counter and calculated apoAI-, HDL-, and serum-mediated cholesterol efflux as previously described [29].

### 2.5. RT-qPCR

We extracted total cellular RNA as previously described [29]. Briefly, cells were rinsed with PBS, and total RNA harvested via a Direct-Zol RNA purification kit (Zymo Research, Irvine, CA, USA). After total RNA isolation, we quantified total RNA with a SpectraMax^®^ QuickDrop™ Micro-Volume Spectrophotometer (MolecularDevices, LLC., San Jose, CA, USA). We used 100 ng of total RNA per sample for cDNA synthesis by converting the RNA into cDNA using a Quantabio qScript^®^ cDNA SuperMix kit (Beverly, MA, USA). We used the cDNA as template for qPCR along with a Quantabio PerfeCTa SYBR Green Fastmix kit for amplifying the following genes: ACTA2 (forward primer, 5′-GCTTCGCTGGTGATGATGCTC-3′; reverse primer, 5′-AGTTGGTGATGATGCCGTGTTC-3′); CD68 (forward primer: 5′-CTTCCCACAGGCAGCACA-3′; reverse primer: 5′-ATGATGAGAGGCAGCAAGAGG-3′); and GAPDH housekeeping gene for normalization (forward primer: 5′-CGTGCCGCCTGGAGAAAC-3′; reverse primer: 5′-TGGGAGTTGCTGTTGAAGTCG-3′). We quantified qPCR reactions using a qTOWER^3^ G touch qPCR machine (Analytik Jena US) and calculated gene expression levels using the delta-delta CT (ΔΔ^CT^) method [36].

### 2.6. Cellular Imaging

We plated MOVAS cells in four-chamber TC slides (Corning) to be used for staining. MOVAS cell staining and imaging was conducted at the Clemson Light Imaging Facility (CLIF), as previously described [29]. Briefly, cells were incubated with mouse anti-ACTA2 (sc-32251; Santa Cruz Biotechnology) and mouse anti-CD68 (sc-20060; Santa Cruz Biotechnology) primary antibodies, then incubated with Alexa Fluor 546 goat anti-mouse IgG_2a_ (Invitrogen, Carlsbad, CA, USA) and Alexa Fluor 488 goat anti-mouse IgG_1_ (Invitrogen) secondary antibodies. We detected cell nuclei via staining with DAPI (Invitrogen). Cellular imaging was performed using a Leica SP8X MP Confocal System equipped with HyD detectors, a 405 nm laser, a tunable white light laser, and time gating capacity (Leica Microsystems, Buffalo Grove, IL, USA). To capture and export images of the stained MOVAS cells, Leica LAS-X software (Leica Microsystems Version 3.5.5.19976) was utilized.

### 2.7. Statistics

SigmaPlot (v14.0) software (Systat Software Inc., San Jose, CA, USA) was used for statistical analyses. For all datasets, normality was determined using a Shapiro–Wilk test and equal variance was assessed by performing a Brown-Forsythe test. For statistical tests involving 2 groups, a Student’s *t*-test was performed if both normality and equal variance were assumed. We performed a Mann–Whitney rank sum test when normality was not assumed and a Welch’s *t*-test when equal variance was violated. For statistical tests involving >2 groups, a one-way ANOVA and Tukey test were performed when normality and equal variance were assumed, while we performed a Kruskal–Wallis one-way ANOVA on ranks and Dunn’s test when equal variance was not assumed. When our studies required statistical analysis, we conducted three independent experiments with 3 biological replicates per experiment, with these data points being represented in our figures and group means denoted as bars. For all statistical tests, we set significance at *p* < 0.05.

## 3. Results

### 3.1. HDL-Mediated Compensatory Cholesterol Efflux Occurs in ABCG1-Deficient MLC

**Figure 1:**
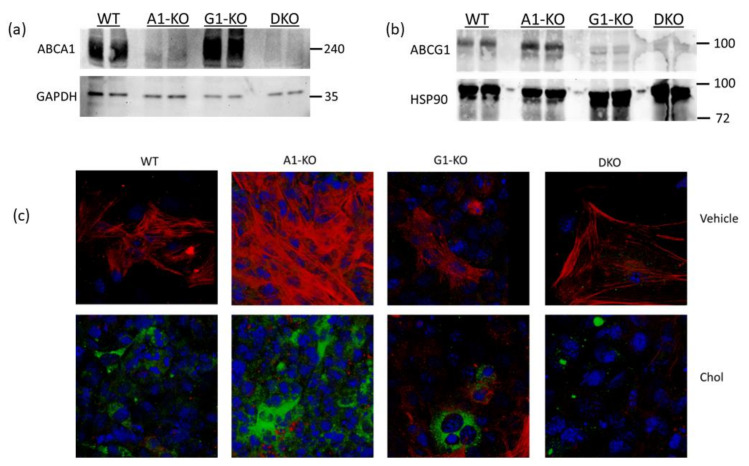
Characterizing ABCA1/ABCG1-deficient MOVAS cells. (**a**,**b**) MOVAS wild-type (WT), ABCA1 KO (A1-KO), ABCG1 KO (G1-KO), or ABCA1/ABCG1-double KO (DKO) cells were treated with GW3965 to attempt induction of ABCA1 and ABCG1 protein expression. Post GW3965 exposure, lysates were used for immunoblotting to probe for ABCA1, ABCG1, or housekeeping proteins (GAPDH and HSP90). (**c**) WT, A1-KO, G1-KO, and DKO MOVAS cells were either incubated with vehicle only or exposed to MβCD:Chol (Chol) to trigger MLC transdifferentiation. Post treatments, cells were stained ACTA2 (red), CD68 (green), and DAPI (blue; cell nuclei) to assess VSMC-to-MLC transdifferentiation. (**a**,**b**) Size markers shown in kDa.

**Figure 2:**
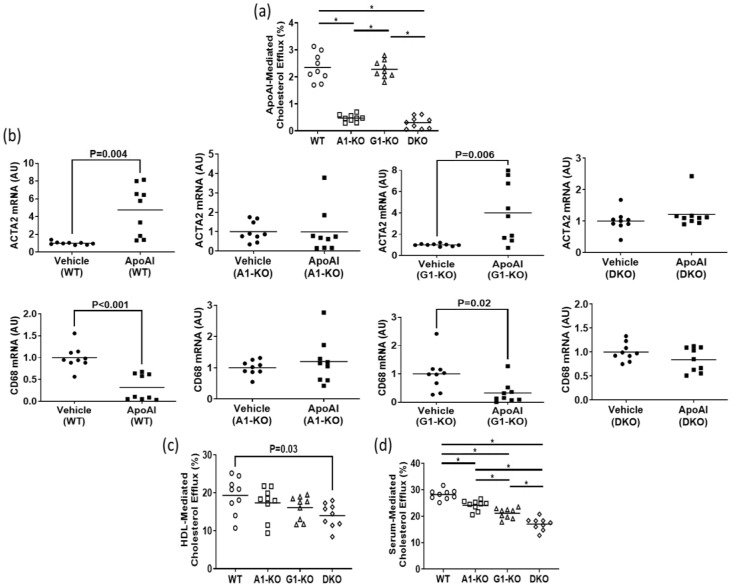
Impact of ABC transporter deletion in MLC of VSMC origin when exposed to cholesterol acceptors. (**a**) ApoAI-mediated cholesterol efflux measured in MOVAS wild-type (WT), ABCA1 KO (A1-KO), ABCG1 KO (G1-KO), or ABCA1/ABCG1-double KO (DKO) MLC. (**b**) ACTA2 and CD68 mRNA expression measured in MOVAS WT, A1-KO, G1-KO, and DKO loaded with MβCD:Chol and then treated with either vehicle only or incubated with apoAI. (**c**,**d**) Cholesterol efflux measured in MOVAS WT, A1-KO, G1-KO, and DKO MLC treated with either HDL (**c**) or serum (**d**). (**b**) AU, arbitrary units. (**a**,**d**) The symbol (*) indicates statistical significance at *p* < 0.05.

Using immunoblotting, we assessed ABCA1/ABCG1 deletion in KO MOVAS cells. We also treated cells with the LXR agonist GW3965 which robustly induces ABCA1/ABCG1 expression [32,33]. The rationale for GW3965 treatment was to attempt to stimulate ABC transporter expression that may be obscured in KO cell populations under normal conditions if cell populations are contaminated with unedited cells that still express endogenous ABCA1/ABCG1. In treated MOVAS cells, we showed the effective ablation of ABC transporter expression in the respective ABCA1/ABCG1 deficient cells (Figure 1a,b). Since edited cells sometimes lose phenotypic properties [37], we analyzed whether ABC transporter-deficient MOVAS cells could still convert into MLC [29,30]. As anticipated, MOVAS KO cells do transdifferentiate into MLC, as determined by the reduced expression of the VSMC marker ACTA2 coinciding with the induced expression of the macrophage marker CD68 [29] (Figure 1c). We proceeded to measure apoAI-, HDL-, and serum-mediated cholesterol efflux in WT and KO MOVAS MLC. We detected a striking decrease in apoAI-mediated cholesterol efflux in ABCA1-deficient MOVAS MLC (Figure 2a). This decrease in apoAI-mediated cholesterol efflux in ABCA1-deficient MOVAS MLC was also associated with an impaired ability to restore VSMC phenotype upon exposure to apoAI, as determined by failing to restore normal expression of the VSMC marker ACTA2 and the failure to suppress the expression of the macrophage marker CD68 (Figure 2b). However, when we measured HDL- and serum-mediated cholesterol efflux in WT and MOVAS KO MLC (Figure 2c,d), the reduction in HDL-mediated cholesterol efflux observed in ABCG1-deficient MOVAS MLC was not as striking when compared to the percent reduction observed in ABCA1-deficient MOVAS MLC for apoAI-mediated cholesterol efflux. Thus, this result implies alternative mechanisms may contribute to HDL-mediated cholesterol efflux other than ABCG1.

### 3.2. SR-BI Protein Upregulation Occurs in ABCG1-Deficient MOVAS MLC

**Figure 3:**
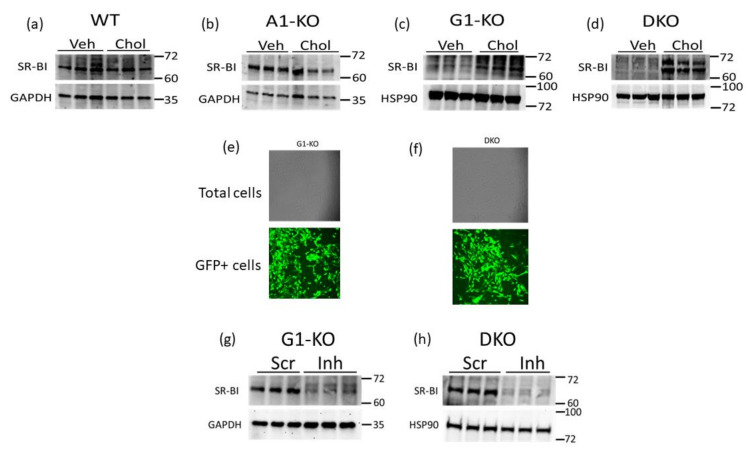
SR-BI induction in MOVAS MLC with ABCG1 deletion. (**a**–**d**) SR-BI protein and GAPDH loading control analyzed via immunoblotting in MOVAS wild-type (WT), ABCA1 KO (A1-KO), ABCG1 KO (G1-KO), and ABCA1/ABCG1-double KO (DKO) treated with vehicle (Veh) only or loaded with MβCD:Chol (Chol) to trigger MLC transdifferentiation. (**e**,**f**) Transduction efficiency assessed via fluorescent microscopy in G1-KO (**e**) and DKO (**f**) MOVAS cells transduced with a GFP-expressing lentiviral vector. (**g**,**h**) SR-BI downregulation detected via immunoblotting in G1-KO (**g**) and DKO (**h**) MOVAS MLC transduced with a lentiviral vector expressing an SR-BI inhibitory shRNA (Inh) when compared to MOVAS MLC transduced with lentivirus expressing a non-targeting scrambled control shRNA (Scr). (**a**–**d**,**g**,**h**) Size markers are kDa.

SR-BI can efflux cholesterol to HDL [22,25,38] and therefore may be a compensatory mechanism for VSMC/MLC to preserve HDL-mediated cholesterol efflux when ABCG1 expression is impaired. Hence, we assessed the SR-BI protein in WT and KO MOVAS cells that were either phenotypically normal (VSMC) or cholesterol-loaded to induce MLC transdifferentiation. Within the MOVAS-WT and MOVAS-A1KO cells, we observed no change in the SR-BI protein when comparing VSMC and MLC protein levels (Figure 3a,b). However, in ABCG1-deficient cells, we observed SR-BI upregulation in MLC when compared to VSMC (Figure 3c,d), potentially indicating that SR-BI is a possible facilitator for maintaining HDL-mediated cholesterol efflux in MLC that have impaired ABCG1 expression. To attempt to manipulate SR-BI expression in MOVAS-G1KO and MOVAS-DKO cells, we evaluated lentiviral transduction in these cells using GFP-expressing lentiviral vectors [39], and showed that lentivirus efficiently transduces these KO cell lines (Figure 3e,f). We then utilized lentivirus to knock down SR-BI in MOVAS-G1KO and MOVAS-DKO cells, which resulted in effective SR-BI protein downregulating (Figure 3g,h).

### 3.3. Inhibiting SR-BI in MOVAS-G1KO MLC Reduces HDL-Mediated Cholesterol Efflux and Halts VSMC Restoration

**Figure 4:**
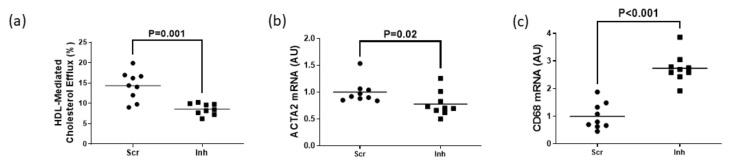
Attenuation of HDL-mediated cholesterol efflux via SR-BI knockdown delays restoring VSMC phenotype in ABCG1-deficient MOVAS MLC. (**a**) HDL-mediated cholesterol efflux measured in ABCG1-deficient MOVAS MLC transduced with a lentivirus either expressing an shRNA directed at SR-BI (Inh) or a non-targeting scrambled control shRNA (Scr). (**b**,**c**) Gene expression of ACTA2 (**b**) and CD68 (**c**) measured in ABCG1-deficient MOVAS-MLC transduced with either Scr or Inh and exposed to HDL to promote cholesterol efflux. AU, arbitrary units.

To assess whether SR-BI plays a role in maintaining HDL-mediated cholesterol efflux in MLC exhibiting impaired ABCG1-dependent cholesterol efflux, we measured HDL-mediated cholesterol efflux in MOVAS-G1KO MLC. To manipulate gene expression in these cells, we transduced MOVAS-G1KO MLC with a lentiviral vector that either expresses a non-targeting scrambled control shRNA or an shRNA that inhibits SR-BI expression. When SR-BI knockdown occurred in MOVAS-G1KO MLC, this decreased HDL-mediated cholesterol efflux when compared to control MOVAS-G1KO MLC (Figure 4a). When we measured mRNA expression of the classical VSMC marker ACTA2 and classical macrophage marker CD68 [29,30] within these groups of cells, we observed that inhibiting SR-BI in MOVAS-G1KO MLC prevents these cells from converting back to their original VSMC phenotype when exposed to HDL (Figure 4b,c). Therefore, these results suggest that inhibiting SR-BI in MLC attenuates HDL-mediated cholesterol efflux which halts VSMC restoration when ABCG1 expression is impaired in these cells.

### 3.4. Knockdown of SR-BI in MOVAS-DKO MLC Prevents VSMC Restoration via Decreasing Serum-Mediated Cholesterol Efflux

**Figure 5:**
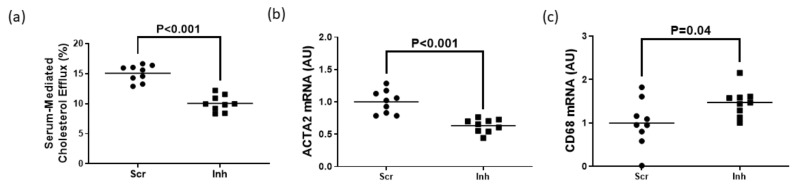
Inhibiting SR-BI in serum-incubated ABCA1/ABCG1 double deficient MOVAS MLC retards VSMC restoration by reducing cholesterol efflux. (**a**) Measuring serum-mediated cholesterol efflux in ABCA1/ABCG1-deficient MOVAS MLC transduced with a lentiviral vector expressing either an shRNA targeting SR-BI (Inh) or non-targeting scrambled control shRNA (Scr). (**b**,**c**) Expression of ACTA2 (**b**) and CD68 (**c**) mRNA measured in ABCA1/ABCG1-deficient MOVAS-MLC exposed to serum and transduced with Scr versus Inh. AU, arbitrary units.

Serum is an endogenous cholesterol acceptor considered more physiologically relevant for measuring cholesterol efflux [24,40] compared to purified apoAI and HDL. However, since serum is abundant in apoAI and HDL [40,41], we measured serum-mediated cholesterol efflux in MOVAS-DKO MLC only to eliminate the possibility of ABCA1/apoAI-dependent cholesterol efflux potentially impacting our results when manipulating SR-BI expression. We observed a significant decrease in serum-mediated cholesterol efflux when knocking down SR-BI in MOVAS-DKO MLC when compared to control MOVAS-DKO MLC (Figure 5a). This reduction in serum-mediated cholesterol efflux hampered these cells’ ability to revert back to their VSMC phenotype (Figure 5b,c). Thus, these findings imply impaired ABC transporter and SR-BI expression in MLC prevent these cells from converting back to their VSMC phenotype, even upon exposure to normal (i.e., serum incubation) physiological conditions.

## 4. Discussion

In this study, we explored the impact of ABC transporter expression on the regulation of cholesterol efflux in MLC. As anticipated, we observed negligible apoAI-mediated cholesterol efflux in ABCA1-deficient MLC. However, while we also expected negligible HDL-mediated cholesterol efflux in ABCG1-deficient MLC, we instead detected reductions that were not as pronounced as we originally anticipated, which led us to investigate other mechanisms responsible for HDL-mediated cholesterol efflux in MLC with impaired ABCG1-dependent cholesterol efflux. In ABCG1-deficient MLC, we observed the upregulation of SR-BI, which indicated a possible mechanism for preserving HDL-mediated cholesterol efflux in ABCG1-deficient MLC. When we inhibited SR-BI in ABCG1-deficient MLC, this decreased HDL- and (HDL-rich) serum-mediated cholesterol efflux, which hindered these cells in reverting to their VSMC phenotype. Taken together, these results suggest impaired ABCA1, but not ABCG1, in MLC negatively impacts cholesterol efflux capacity, as SR-BI preserves HDL-mediated cholesterol efflux when ABCG1-dependent cholesterol efflux is impaired.

Emerging evidence indicates VSMC are a major cell type that influences atherosclerosis development [42,43]. Furthermore, cholesterol accumulation in VSMC induces MLC transdifferentiation, which plays a pivotal role in atherosclerosis progression [44,45]. Therefore, strategies to increase cholesterol removal in MLC may be a potential therapy for atherosclerosis. Since ABCA1 and ABCG1 are two transporters primarily responsible for removing excess cholesterol in peripheral cells [46], upregulating the expression of these two transporters in VSMC/MLC may be a promising atheroprotective approach. Research has shown that MLC of VSMC origin have decreased ABCA1 expression [11,12,47], though there has been little focus on assessing the atherogenic impact of ABCG1 expression in VSMC/MLC [48]. This is the only study, to the authors’ knowledge, that has directly tested the impact of ABCG1 deficiency on cholesterol efflux in cultured MLC. This work uncovered the fact that ABCG1 deletion in MLC does not entirely diminish the ability of these cells to efflux cholesterol to HDL, as we discovered SR-BI induction in MLC compensates for the lack of ABCG1 to preserve HDL-mediated cholesterol efflux. From this observation, we infer that at least in VSMC/MLC, SR-BI plays more than a minor supportive role in total cholesterol efflux when ABCG1 expression becomes impaired.

Within this report, we want to highlight some limitations of our study. One drawback is the use of immortalized cells instead of primary cells, as data generated from using immortalized cells is not considered as physiologically relevant in comparison to primary cells [49]. Since ABCA1 and ABCG1 floxed mice are commercially available [50], future studies should involve utilizing Cre-Lox technology and ex vivo strategies [51] to test whether our findings can be reproduced when primary VSMC are used. Another drawback to our experiments is that we did not attempt to identify the precise mechanism(s) underlying what induces SR-BI protein expression in MLC that exhibit impaired ABCG1. Discovering what controls this process may lead to therapies geared at robustly increasing SR-BI expression in VSMC/MLC and other cell types, which may eventually be used as a form of treatment for atherosclerosis. Lastly, we did not manipulate SR-BI expression in VSMC/MLC that demonstrated native ABCG1 expression, as it would be interesting to test if deletion of SR-BI in MLC results in upregulation of endogenous ABCG1 to compensate for defective SR-BI expression. Thus, future studies should analyze whether HDL-mediated cholesterol efflux in MLC is negatively impacted by impaired SR-BI or if ABCG1 expression is induced to compensate for a loss of SR-BI-dependent cholesterol efflux.

**Figure 6:**
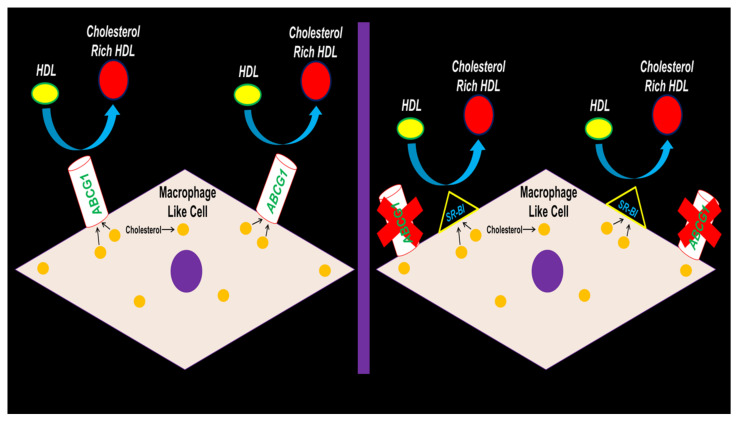
Proposed working model depicting SR-BI upregulation maintaining HDL-mediated cholesterol efflux in MLC of VSMC origin when ABCG1 expression becomes impaired.

In conclusion, our results depict SR-BI preserving HDL-mediated cholesterol efflux in MLC when ABCG1 expression is hampered (Figure 6). Future studies should be devoted to testing if ABCG1 deletion within the VSMC of atherogenic mouse models results in exacerbating atherosclerosis. If results show that atherosclerotic lesion size and lipid content does not change from VSMC ABCG1 deficiency in vivo, then VSMC SR-BI expression/function should be assessed to deduce whether SR-BI induction in VSMC arising from VSMC ABCG1 deletion confers protection against atherosclerosis progression by preserving VSMC/MLC HDL-mediated cholesterol efflux.

## Data Availability

All the data within this study are provided in the manuscript.
